# Cities without Flies

**Published:** 1916-03

**Authors:** 


### Cities Without Flies.

The press everywhere is calling attention to the fact that it is possible to have cities without flies. It has only been a few years since flies were regarded as a necessary evil from which there was no hope of permanent escape. Then the "swat the fly" campaigns were started with the idea of reducing the number and thereby decreasing the bad results coming from the pests. Now it has been found that with anything like concert of action flies can be exterminated in any city, and that all that is required is to inaugurate campaigns for cleanliness.

A few years ago there was not a worse pest hole in the world than Havana. It was loathsome in its filth, and the Cubans were regarded as hopelessly filthy in their habits. Yellow fever and other contagious and epidemic diseases were regarded as things that had to be endured, because they could not be remedied. The United States government led in a campaign for cleaning Havana and ridding it of flies and mosquitoes, the result being that Havana has been made the cleanest city and therefore the healthiest city on the globe. The Cubans have come to realize that cleanliness is so closely related to healthfulness that they cannot afford to be unclean.

What Havana has done every city, every village, every home in the land can do. But to bring it about a campaign of education will be required, and in this campaign the physicians will be looked to for leadership. The time is not far distant when to see a town infested with flies and mosquitoes will be considered a reflection upon the physicians of that town because of their lack of attention to sanitary measures. Already the clean town is regarded as a place in which the local physicians interest themselves in sanitary measures, and the dirty, filthy town is put down as the home of indifferent physicians. In which class do you prefer to be placed?

